# Post-splenectomy Sepsis: A Review of the Literature

**DOI:** 10.7759/cureus.6898

**Published:** 2020-02-06

**Authors:** Faryal Tahir, Jawad Ahmed, Farheen Malik

**Affiliations:** 1 Internal Medicine, Dow University of Health Sciences, Karachi, PAK; 2 Pediatrics, Dow University of Health Sciences, Karachi, PAK

**Keywords:** splenectomy, spleen, overwhelming post-splenectomy infection, opsi, sepsis, vaccination, asplenia, infection

## Abstract

The spleen is an intraperitoneal organ that performs vital hematological and immunological functions. It maintains both innate and adaptive immunity and protects the body from microbial infections. The removal of the spleen as a treatment method was initiated from the early 1500s for traumatic injuries, even before the physiology of spleen was properly understood. Splenectomy has therapeutic effects in many conditions such as sickle cell anemia, thalassemia, idiopathic thrombocytopenic purpura (ITP), Hodgkin’s disease, and lymphoma. However, it increases the risk of infections and, in some cases, can lead to a case of severe sepsis known as overwhelming post-splenectomy infection (OPSI), which has a very high mortality rate. Encapsulated bacteria form a major proportion of the invading organisms, of which the most common is *Streptococcus pneumoniae*. OPSI is a medical emergency that requires prompt diagnosis (with blood cultures and sensitivity, blood glucose levels, renal function tests, and electrolyte levels) and management with fluid resuscitation along with immediate administration of empirical antimicrobials. OPSI can be prevented by educating patients, vaccination, and antibiotic prophylaxis. This article summarizes the anatomy and physiology of the spleen and highlights its important functions. It primarily focuses on the pathophysiology of OPSI, its current management, and prevention strategies.

## Introduction and background

The spleen, a lymphoid aggregate, lies behind ribs 9-10 in the left hypochondriac region of the abdominal cavity where a part of it lies in the epigastric region. Thus, the largest organ of the lymphatic system is situated between the fundus of the stomach and the diaphragm. Being a highly vascular structure, the spleen appears spongy and reddish-purple in color. An adult human spleen is roughly the size of a clenched fist, although its size and weight vary, measuring about 10-12 cm (3.9-4.7 inch) vertically in the longest dimension while weighing 150-200 grams on average [1,2]. It is the only lymphoid organ that lies directly in the path of the blood circulation. Due to its heterogeneous structure, the spleen performs a variety of immunological and hematological functions. It plays a key role in both the innate and adaptive immune systems, thereby protecting the body from invading organisms [3].

Therapeutic splenectomy for malarial splenomegaly was performed as early as 1549 by Adriano Zaccaria. The first partial splenectomy dates back to 1590 and was performed by Franciscus Rosetti for war-related traumatized spleen [4]. In 1826, Quittembaum performed splenectomy in a 22-year-old woman for the removal of a large splenic tumor. In 1893, Jaboulay coined the term “exosplenopexy” to describe the partial removal of the spleen while attaching it to the external wound edges. The exterior part of the spleen would then eventually undergo atrophy. In comparison, Pallavecchio destroyed the exterior part of the spleen thermally instead of waiting for spontaneous regression and, hence, named the process “exosplenolisi” [5].

In 1911, Emil T Kocher stated that splenic injuries required the removal of the whole organ, and it had no adverse effects [4]. This led to the belief that splenectomy could be performed in all cases. However, in 1919, Morris and Bullock established that partial or complete removal of the spleen was associated with an increased risk of infections in splenectomized individuals [3]. This finding was further supported by King and Shumacker in 1952 when they reported severe sepsis in five children who had undergone splenectomy for congenital hemolytic anemia [6]. In 1962, Horan and Colebatch published a detailed review that revealed that incidences of fatal infection in asplenic children were 10 times higher (5.6-8.7%) than in the control population (0.7%) [7]. Thus, the removal of the spleen makes patients susceptible to a variety of different infections caused by organisms such as *Staphylococcus aureus*, *Haemophilus influenzae*, *Streptococcus pneumoniae*, and malarial parasites. Sepsis in asplenic patients is more likely to be caused by encapsulated organisms as they are more resilient to phagocytosis. The most dreadful complication of splenectomy is overwhelming post-splenectomy infection (OPSI), which is associated with significant morbidity and mortality rates.

Scientific community and physicians are using a multidimensional approach to tackle the problem of OPSI. This approach involves patient education, the use of preoperative as well as postoperative vaccinations against specific organisms, prophylaxis with antibiotics, and the maintenance of a spleen registry following splenectomy. We aimed to review the existing literature on the topic and gather a comprehensive picture of splenectomy and the associated sequelae along with its management and preventive strategies.

## Review

The Spleen

Embryology

During the sixth week of fetal development, the spleen develops from the left layer of dorsal mesogastrium in the cephalic part into multiple nodules that later fuse to form a lobulated spleen. In an adult spleen, notching of the superior border shows evidence of its multiple origins. During intrauterine life, the rotation of the stomach causes fusion of the left mesogastric surface with the peritoneum above the left kidney and, consequently, dorsal attachment of the lienorenal ligament. Cells required for the hematopoietic function of the spleen are supplied by the yolk sac and dorsal aorta. By the second trimester, the spleen is able to generate red and white blood cells into the blood circulation.

Location

The spleen lies posterior to the stomach and anterior to the left hemidiaphragm in the left hypochondriac region of the abdominal cavity, relatively below the left costal margin. It rests on the left colic (splenic) flexure, facing the left kidney and pancreatic tail medially and the diaphragm superiorly. Although it can descend up to pubic symphysis in certain disorders, a healthy normal spleen is usually not palpable in most individuals as it normally does not extend beyond the rib arch.

Gross Anatomy

The spleen is encased in a weak fibroelastic connective tissue capsule that offers protection and expansion. The capsule further subdivides into smaller internal sections known as lobules. Being an intraperitoneal organ, all surfaces of the spleen are enveloped in the visceral peritoneum, except the hilum, which incorporates nerves, splenic vessels, and ligamentous attachments. The spleen has two surfaces: the diaphragmatic or lateral surface (smooth and convex) and the visceral or medial surface (concave and irregular with several imprints). The diaphragmatic surface shows imprints from ribs 9-11, while the visceral surface shows imprints from left colic flexure (colic area), stomach (gastric area), and left kidney (renal area). The spleen has three borders: superior (which bounds the gastric area), inferior (which bounds the renal area), and anterior (which bounds the colic area). The splenic hilum is found inferomedial to the gastric area. The pancreatic tail leaves an impression between the hilum and colic area. Three ligaments originating from adjacent viscera connect with the spleen. Out of them, two ligaments approach the splenic hilum as they are traversed by the transmitted vascular supply of the spleen. These ligaments include the gastrosplenic ligament (which connects splenic hilum with greater gastric curvature) and the splenorenal ligament (which bridges splenic hilum with left kidney). The latter transmits splenic artery and vein whereas the former contains short gastric vessels and left gastroepiploic arteries and veins. Lastly, the spleen is supported by the phrenicocolic ligament, which takes its origin from the colon and is regarded as sustentaculum lienis.

Microscopic Anatomy and Functions of the Spleen

**Figure 1:**
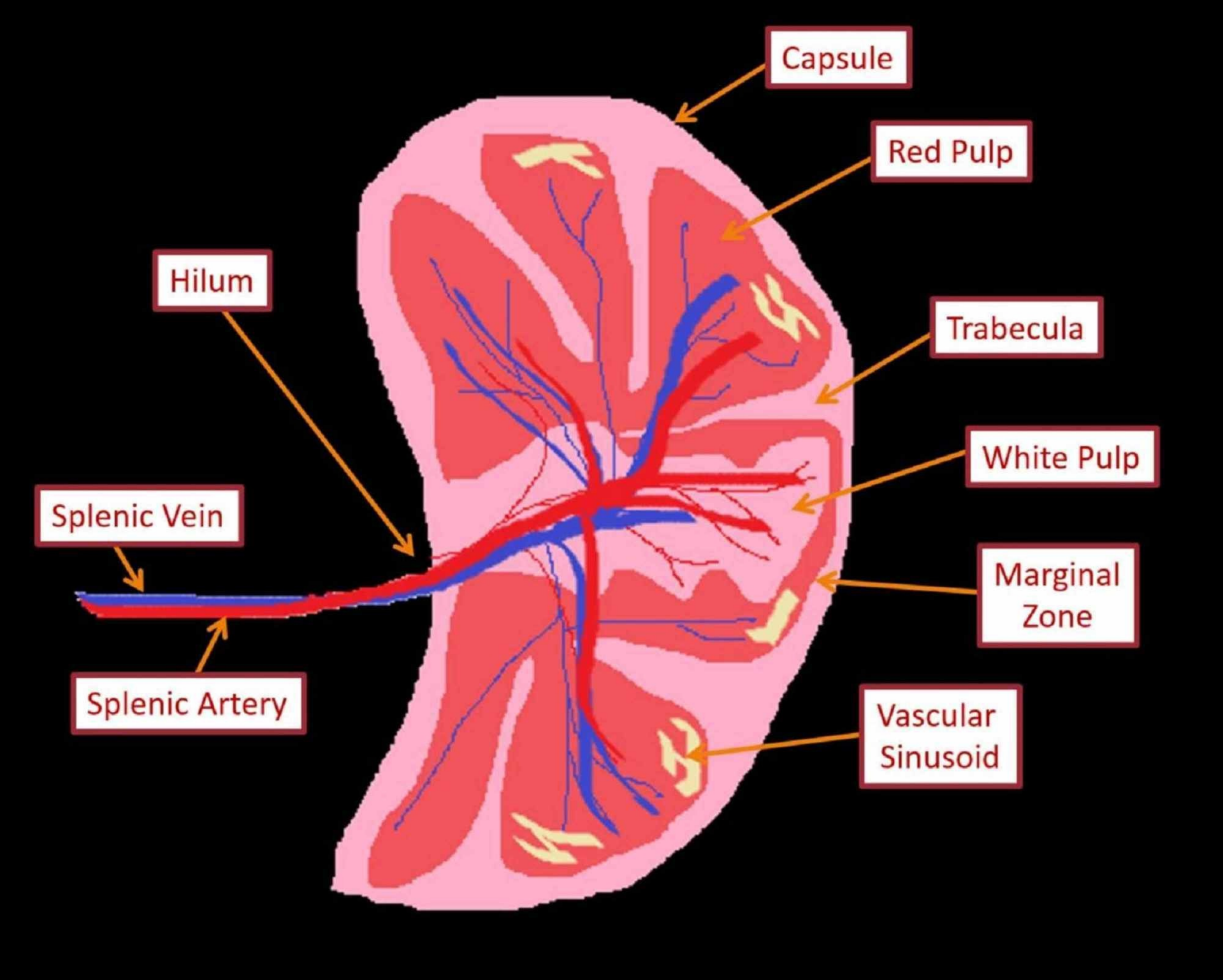
Schematic diagram of microstructure of the spleen

Knowledge of the microscopic anatomy of the spleen is vital for the understanding of its functions. The dense irregular connective tissue of the capsule gives numerous septa called trabeculae that extend into the parenchyma of the spleen. Myoepithelial cells are found in the capsule as well as in the trabeculae, which contract to pump stored blood into the circulatory system whenever the body is in need, i.e., during intense physical activity or severe hemorrhage. The parenchyma of the spleen (pulp) contains two different types of tissues, termed white and red pulp, each with unique functions. The white pulp, constituting 25% of the total splenic volume, is primarily lymphocytic. It is mainly composed of periarteriolar lymphoid sheaths (PALS; T-cell dominant) and lymphatic nodules (B-cells). The remaining 75% of the splenic volume is occupied by the red pulp, which incorporates splenic venous sinuses, cords (of Billroth), and perisinusoidal macrophages. Separating white and red pulp is the marginal zone that filters pathogens out of the blood and presents them to the lymphocytes residing in white pulp [8]. A schematic diagram of the microstructure of the spleen is shown in Figure 1. Table 1 summarizes the neurovascular supply and lymphatic drainage of the spleen. Table 2 enlists various functions executed by a healthy human spleen.

**Table 1:** Neurovascular supply and lymphatic drainage of the spleen

Characteristics	Structures involved
Arterial supply	Splenic artery, which further divides into five branches that do not anastomose, giving rise to vascular segmentation of the spleen
Venous drainage	Splenic vein, which later joins superior mesenteric vein to give rise to the portal vein
Innervation	Celiac plexus (sympathetic and parasympathetic)
Lymphatic drainage	Celiac lymph node (receives lymph from pancreaticosplenic lymph nodes)

**Table 2:** Functions of the spleen IgM: immunoglobulin M

Immunological functions	Hematological functions
Red pulp macrophages decontaminate the blood from pathogens	Removal of unwanted intra-erythrocytic inclusions, i.e., Howell–Jolly bodies and erythrocyte pits
Marginal zone macrophages get rid of the circulating microorganisms and cellular debris	Pooling of platelets along with sequestration of erythrocytes, granulocytes, plasmablasts, and plasma cells
Tingible body macrophages discard B-cell debris in the germinal center of the follicle	Phagocytosis of defective or old erythrocytes in the blood circulation
Dendritic cells, natural killer cells, and monocytes induce T lymphocyte formation	Hematopoiesis during fetal life
Splenic B cells produce antigen-specific antibodies and augment cytotoxic T cell response	Extramedullary hematopoiesis (if required)
IgM memory B cells produce IgM to promote the clearance of polysaccharide-encapsulated bacteria	Storage of iron
Production of immune mediators such as complement, opsonins, properdin, and tufts, which instigate phagocytosis	

Changes in Splenic Function with Advancing Age

Inadequate defense mechanism against encapsulated bacteria during the early and later years of life is a consequence of a natural process involving splenic cellular maturation, which occurs during the first two decades of life. The concentration of follicles diminishes as individual ages, particularly after the third decade, until they are atrophic by the eighth decade of life. This indicates that the immunological function of the spleen deteriorates with age [9].

Splenectomy

Splenectomy, or the surgical removal of the spleen, is usually considered a life-saving procedure in traumatized individuals, certain hematological disorders, malignant conditions, and for the sake of diagnostic purposes [10]. Globally, the incidence of splenectomy is nearly 6.4-7.1 per 100,000 people per annum, with trauma (25%) and hematological disorders (25%) being the most common culprits [5]. Abdominal trauma leading to the rupture of the spleen with internal bleeding and hemodynamic instability usually ends up with splenectomy [11]. But, due to the advent of alternative approaches, splenectomy in trauma settings is on the decline. Hematological conditions requiring splenectomy include idiopathic thrombocytopenic purpura (immune thrombocytopenia; ITP), sickle cell disease, thalassemia, and hereditary spherocytosis (HS) [6,12,13]. Table 3 lays out the main indications and contraindications of splenectomy.

**Table 3:** Indications and contraindications for splenectomy HS: hereditary spherocytosis; TB: tuberculosis; ITP: idiopathic thrombocytopenic purpura

Absolute indications for splenectomy	Relative indications for splenectomy	Absolute contraindications for splenectomy	Relative contraindications for splenectomy (laparoscopic approach)
Splenic trauma, i.e., splenic rupture either spontaneous (tropical splenomegaly) or delayed (subcapsular hematoma)	Congenital or acquired hemolytic anemia	Uncorrectable coagulopathy	Active hemorrhage with hemodynamic instability
HS	Thalassemia	Severe cardiovascular disease making the patient unfit for general anesthesia	Non-platelet coagulopathy
Splenic abscess (TB infection)	Acute, chronic myeloid or chronic lymphatic leukemia	Cirrhosis with portal hypertension	Contraindications to pneumoperitoneum
Splenic cyst	Lymphoma (Hodgkin’s)		Splenomegaly
As part of radical surgical removal of locally advanced gastric carcinoma, pancreatic carcinoma	Polycythaemia vera and myelofibrosis		Pregnancy
Angioma	Acute or chronic ITP		Extensive previous upper abdominal surgery
Primary splenic malignancy (rare)	Parasitic infestation, malaria, and Felty’s syndrome		
Aneurysm of splenic artery	Angioma, cysts, and metastases		
Bleeding esophageal varices secondary to splenic vein thrombosis	Tropical or non-tropical splenomegaly		
	Intrahepatic or extrahepatic portal hypertension		
	Amyloidosis and Gaucher’s disease		

Infections Following Splenectomy

Although the presence of spleen is not imperative for the survival of human beings, its removal still has consequences. Until 1952, when King and Schumacher revealed the risk of OPSI, the spleen was considered an unnecessary organ and a spleenless existence was thought to be quite safe [5]. Nowadays, it is clearly evident that splenectomy induces an increased risk of infection and thromboembolism [14]. The factors responsible for an increased incidence of infection and propensity for severity following a splenectomy include insufficient opsonizing filter function of the spleen, delayed and impaired production of immunoglobulin (Ig), lack of splenic macrophages, and minimal tufts production [5]. Nevertheless, the complement system remains unchanged as levels of serum complement components C3, C4, and transferrin do not fluctuate [15]. Furthermore, the abolishment of the cholinergic anti-inflammatory pathway ensuing splenectomy may contribute to the increased propensity for OPSI, although a direct association has not been established yet [5].

Splenectomy can be immediately followed by reactive thrombocytosis and leukocytosis. The former usually resolves within 6-12 months, whereas the latter may persist for many years following the removal of the spleen [16]. Leukocytosis is predominantly granulocyte-driven, as elevated levels of neutrophils are usually appreciated after splenectomy [17]. Along with the quality of erythrocytes, the proportions of lymphocyte cohorts are also altered. Although the total B lymphocytes remain intact, a significant fall in the levels of memory B cells and switched B cell proportions are usually encountered 150 days post-splenectomy [18]. This acts as a particular predisposition to infections caused by polysaccharide-encapsulated bacteria and is responsible for a diminished immunological response to polysaccharide vaccines [19,20]. These hematological and immunological amendments predispose splenectomized individuals to various infections. Following splenectomy, individuals end up with an elevated risk of infection, mainly from encapsulated Gram-negative pathogens, i.e., *Capnocytophaga canimorsus* and *Bordetella holmesii* [21, 22], and intraerythrocytic parasites, i.e., intraerythrocytic malaria and *Babesia* parasites [23,24].

Overwhelming post-splenectomy infection

The major long-term complication of splenectomy is OPSI, also known as post-splenectomy sepsis syndrome, which is defined as a generalized non-specific flu-like prodrome followed by rapid deterioration to full-blown fulminant septic shock within 24-48 hours of the onset [9]. Although there is no specific diagnostic criterion for OPSI, prompt identification followed by proper management can prevent further deterioration and fatality [25]. The prevalence of OPSI following splenectomy is 0.1-0.5%, with a mortality rate of up to 50% [26].

The period of highest risk for infections is during the first 3 years post-splenectomy; however, the risk remains elevated throughout an individual’s lifespan, indicated by the reported occurrence of cases of fulminant infection 20 years after splenectomy [6]. Children under 2-5 years of age, those who have had splenectomy post-trauma, individuals splenectomized for hematological malignancy or malignant conditions, and immunosuppressed or immunodeficient individuals (e.g., HIV-infected) are at a greater risk for OPSI [5,27].

In the late 1990s and early 2000s, pneumococcus was considered the predominant cause of infection post-splenectomy (57-87%). However, recent studies suggest that *Neisseria meningitidis* and *Haemophilus influenzae* (type b) are also common etiologic agents [28,29]. Less common organisms include Gram-negative bacteria such as *Pseudomonas aeruginosa*, *Capnocytophaga canimorsus*, *Bartonella* spp., and *Babesia* spp. [30]. Pneumococcal infection is by far the most common with an associated mortality rate of up to 60% [20,24]. However, reports from Denmark have shown *Escherichia coli (E. coli)* to be the most prevalent in post-splenectomy bacteremia; but it may be a regional phenomenon [25]. This might relate to the concomitant administration of penicillin and pneumococcal vaccine in splenectomized individuals [31].

OPSI is a medical emergency as rapid cardiovascular collapse and death have been reported to occur within 12-24 hours of the onset of symptoms [29]. It starts as a prodrome with fever, chills, myalgia, headache, vomiting, and abdominal pain, progressively leading to coma, septic shock, and disseminated intravascular coagulation (DIC) [32]. Polysaccharide-specific antibodies activate the complement pathway, thereby promoting the deposition of complement fragments directly on to the capsule and, hence, thrombotic vascular occlusion. This might suggest an association between OPSI and DIC [33]. An indication for the administration of corticosteroids in OPSI is a frequent finding of bilateral adrenal hemorrhage, mimicking Waterhouse-Friderichsen syndrome (WFS) [29].

Initially, the condition may be difficult to diagnose. However, any splenectomized individual with symptoms like fever, chills, diarrhea, and vomiting should be assessed for OPSI. Individuals suffering from severe sepsis or septic shock must be evaluated in accordance with the international guidelines [34] along with prompt administration of empiric, broad-spectrum antibiotics. At least two sets of blood cultures should be collected prior to antibiotic therapy for the identification of the offending pathogen. Laboratory investigations should cover blood glucose level, serum lactate concentration, and electrolytes with hematological and renal profiles. A peripheral blood smear or a buffy coat for the presence of bacteria and a blood film for the evaluation of Howell-Jolly bodies should be urgently ordered while awaiting the results of blood cultures. Evidence shows that the instantaneous administration of empiric antimicrobial therapy is associated with an increased survival rate [35]. Additionally, aggressive fluid therapy and constant monitoring also play an imperative role in the management of OPSI. In order to diminish the levels of inflammatory mediators and enhance the hemodynamic stability in splenectomized individuals with pneumococcal sepsis, it has been proved beneficial to consider blood purification techniques with adjunctive adsorbent therapy [36,37].

Prevention of post-splenectomy infection

Despite adequate treatment, the mortality rate pertaining to OPSI remains high. This highlights the importance of implementing preventive strategies in the effective management of splenectomized individuals. The British Committee for Standards in Haematology has set guidelines for the prevention and treatment of infections in the asplenic or hyposplenic population, which can be divided roughly into three categories: patient education, vaccination, and prophylaxis with antibiotics [28].

Patient Education

A vast majority (85%) of splenectomized individuals are not aware of their increased susceptibility to infectious diseases and the need to take relevant health precautions [38]. Inadequate information and lack of sufficient education seem to be the major culprit behind this lack of awareness [36,39]. Patients and their family members should be educated regarding their asplenic status in both written and electronic form [35]. Table 4 describes the information that must be provided to asplenic individuals.

**Table 4:** Information and directions for splenectomized patients OPSI: overwhelming post-splenectomy infection

Summary of patient education details
Splenectomy carries a lifelong increased risk of infections
Initial symptoms of OPSI include high-grade fever (>38 °C), chills, myalgia, headache, vomiting, and abdominal pain
Please notify your healthcare workers about your asplenic status
Seek immediate medical attention on events like animal bites and scratches
Consider seeking medical advice prior to traveling, especially before visiting a malaria-endemic area
Always carry antibiotic supplies with you (may find helpful in case of sudden illness)
Never forget to carry a medical alert
If available, must consider registering in a “spleen registry system”

Vaccination

All asplenic individuals must receive pneumococcal, *Haemophilus influenza* type b (Hib), meningococcal, and annual influenza vaccinations [35]. These vaccines are mainly recommended for infection prophylaxis due to their clinical effectiveness. For instance, immunization seems to be partially responsible for reducing the rate of post-splenectomy sepsis [40,41]. Keeping in view the Centers for Disease Control and Prevention (CDC)-recommended adult immunization schedule, Table 5 details vaccination guidelines for splenectomized individuals [42].

**Table 5:** CDC-recommended adult immunization schedule after splenectomy CDC: Centers for Disease Control and Prevention; IM: intramuscular

Recommended vaccine	Dose of the vaccine	Route of administration	Time of administration
In-hospital protocol			
Pneumococcal 13-valent conjugate (PCV13: Prevnar 13)	0.5 mL	IM	On the day of discharge or day 14, whichever comes first
Haemophilus influenza type b vaccine (Hib: ActHIB)	0.5 mL	IM	On the day of discharge or day 14, whichever comes first
Meningococcal vaccine (Menactra)	0.5 mL	IM	On the day of discharge or day 14, whichever comes first
Meningococcal serogroup B (Bexsero)	0.5 mL	IM	On the day of discharge or day 14, whichever comes first
Short-term follow-up			
Pneumococcal polysaccharide (PPSV23: Pneumovax 23)	0.5 mL	IM	Two months after the initial vaccination
Meningococcal vaccine	0.5 mL	IM	Two months after the initial vaccination
Meningococcal serogroup B	0.5 mL	IM	Two months after the initial vaccination
Long-term follow-up			
Pneumococcal polysaccharide	0.5 mL	IM	5 years after the first dose
Meningococcal vaccine	0.5 mL	IM	Every 5 years
Seasonal influenza vaccine	-	-	Annually

Antibiotic Prophylaxis

In the 1980s, two controlled trials evaluated the effectiveness of prophylactic penicillin in the pediatric population with sickle cell disease where rates of infection with *Streptococcus pneumoniae* declined [43,44]. Oral penicillins, normally the prophylactic drugs of choice, are now considered less effective due to the emission of bacterial resistance [45]. In contrast, a broader spectrum of activity has been suggested for amoxicillin-clavulanic acid, trimethoprim-sulfamethoxazole (TMP/SMX), or cefuroxime [46]. Initially, after splenectomy, most guidelines recommend a daily regimen of antibiotic therapy, while the need for lifelong consumption can be decided after evaluating the risk of infection [5]. Table 6 enlists individuals who are considered high-risk populations who should be administered lifelong prophylactic antibiotic therapy as recommended by the British guidelines [35].

**Table 6:** High-risk population who should be administered lifelong antibiotic prophylaxis after splenectomy PPV: pneumococcal polysaccharide vaccine

High-risk population
Children younger than 16 years of age
Adults older than 50 years of age
Individuals with a past history of invasive pneumococcal infection
Individuals splenectomized for hematologic malignant diseases, malignant neoplasms, and thalassemia
Individuals in the first year post-splenectomy, irrespective of the cause
Patients with sickle cell anemia
Individuals with a poor response to PPV-23

Benefits of Having a Spleen Registry

In a cross-sectional survey, Wang J. et al. reported higher rates of adherence to current post-splenectomy guidelines among patients who had been registered in the Victorian Spleen Registry. They also reported reduced incidences of infections among this cohort [47]. In another retrospective cohort study, Arnott A. et al. concluded that systemic, long-term approaches can significantly minimize the risk of infection with encapsulated pathogens among splenectomized individuals [48]. According to Premawardena C et al., major benefits of the registry were not related to the knowledge but in the delivery of the recommended vaccines and the use of a medical alert card [49].

## Conclusions

Therapeutic removal of the spleen is currently widely carried out in hematological and oncological disorders. The removal of the spleen leaves the body defenseless against many infections as the body’s ability to opsonize bacteria is weakened. The individual becomes susceptible to a variety of respiratory, urinary, and meningeal infections. Seeding of bacteria from infection sites can lead to bacteremia and a severe case of sepsis knows as OPSI. Due to its rapid progression and high mortality rate, OPSI is considered a serious medical emergency. The timely identification of OPSI, its immediate management, and vigilant care of splenectomized patients provides them with a better chance of survival.

In order to reduce post-splenectomy infections, patient education, vaccination, and prophylactic antibiotics are of prime importance. Splenectomized patients who are aware of the dangers that infections could cause are likely to be more careful and adherent to precautionary measures. All asplenic individuals must receive pneumococcal, *Haemophilus influenza* type b (Hib), meningococcal, and annual influenza vaccinations along with prophylactic antibiotics (amoxicillin-clavulanic acid, TMP/SMX, or cefuroxime) as these measures have been found effective in decreasing the incidence of post-splenectomy infections. The formation of a spleen registry has also been found beneficial in reducing infection rates. Further research is needed to improve the current prevention and treatment strategies for OPSI and thereby improve the quality of life of high-risk (splenectomized or asplenic) patients.
